# Enhancing African horse sickness virus detection: comparing and adapting PCR assays

**DOI:** 10.1177/10406387261417355

**Published:** 2026-02-07

**Authors:** Lisa Penzhorn, Jan E. Crafford, Alan J. Guthrie

**Affiliations:** Equine Research Centre, Faculty of Veterinary Science, University of Pretoria, Onderstepoort, South Africa; Department of Veterinary Tropical Diseases, Faculty of Veterinary Science, University of Pretoria, Onderstepoort, South Africa; Equine Research Centre, Faculty of Veterinary Science, University of Pretoria, Onderstepoort, South Africa

**Keywords:** African horse sickness, reverse transcription real-time PCR, test accuracy

## Abstract

African horse sickness (AHS) is the only equine disease for which the World Organisation for Animal Health (WOAH) gives official disease-free status, given that it poses a major threat to the equine industry. The disease is caused by AHS virus (AHSV; family *Sedoreoviridae*, taxon species *Orbivirus alphaequi*), which is endemic in sub-Saharan Africa. Reverse-transcription quantitative real-time PCR (RT-qPCR) is a rapid, sensitive detection method used in the diagnosis of AHS and the certification of animals as negative for AHSV for the purpose of movement. Genetic variability of AHSV may influence the accuracy of RT-qPCR detection methods because of possible mispriming and/or probe binding failures. We evaluated the diagnostic accuracy of the current WOAH-recommended RT-qPCR assays for the detection of AHSV, namely the Agüero et al. and Guthrie et al. methods. Utilizing 150 AHSV-positive diagnostic samples, we performed in vitro analysis using both assays. The Agüero assay failed to detect AHSV in 13 samples (8.7% false-negative rate). The AHSV VP7 genes of the 13 negative samples, and publicly archived sequences were used to perform in silico analysis, and we incorporated minor changes into the primers and probes of modified Guthrie and modified Agüero assays. A second in vitro analysis yielded 100% sensitivity for both assays. Differences in both the in silico and in vitro analyses highlight the need for continuous monitoring of the efficacy of molecular protocols used for the detection of AHSV.

African horse sickness virus (**AHSV**; family *Sedoreoviridae*, taxon species *Orbivirus alphaequi*) is a double-stranded RNA virus. African horse sickness (**AHS**) is a non-contagious disease of equids that is transmitted by *Culicoides* midges (*Diptera: Ceratopogonidae*) and is usually seen in 1 of 4 clinical forms (i.e., pulmonary, cardiac, mixed, horse sickness fever). Nine antigenically distinct types of AHSV have been identified.^15,21^ In naïve populations of horses, mortality can reach 95%.^6,7,11^ Given the potential for rapid spread and high mortality, AHS is a World Organisation for Animal Health (WOAH)-listed disease.^27^ AHSV is endemic to sub-Saharan Africa but has previously spread to Europe,^24^ the Middle East,^2,14^ and, most recently, to Thailand.^5,7,19,20^ Repeated incursions of various types of the closely related bluetongue virus into Europe,^22^ and recent outbreaks in France and other European countries,^3^ have raised concerns about the potential spread of AHSV to non-endemic areas.

Techniques available for AHSV detection include virus isolation,^13^ ELISA,^9,25^ and reverse-transcription PCR (RT-PCR).^1,12,23^ Rapid, accurate detection of AHSV is essential for prompt diagnosis in an outbreak situation and is necessary to implement appropriate control measures. False-negative assay results could have a devastating effect on the equine industry in non-endemic countries. Accurate surveillance in endemic countries is also essential to identify contemporary field viruses. In 2015, the WOAH Reference Laboratories for AHS assessed the performance of 10 RT-PCR methods used for the detection of AHSV.^28^ The reverse-transcription quantitative real-time PCR (RT-qPCR) assays of Agüero et al.^1^ (hereafter **Agüero assay**) and Guthrie et al.^12^ (hereafter **Guthrie assay**), targeting the highly conserved VP7 gene, had high sensitivity and correctly detected all strains included in the trial. Both methods are validated for certifying individual animals before movement.^28^

Field strains of AHSV are genetically diverse because both genetic drift and reassortment of individual gene segments occur during virus replication in vertebrate and invertebrate hosts.^4,8,23^ Any changes in sequence complementarity within the primer- and probe-binding regions in a specific assay may result in decreased diagnostic sensitivity and/or specificity of the assay. In silico analysis is a simple tool to identify potential issues with detection assays, but the relevance of the analysis hinges on the incorporation of epidemiologically relevant field strains of AHSV. In the case of AHSV, failure to correctly detect the virus, and subsequently the movement of infected horses from an endemic to a non-endemic area, could have catastrophic consequences.

We assessed the performance of the Guthrie and Agüero assays both in silico and in vitro. Based on the in silico analysis, we modified the Guthrie and Agüero assays by incorporating minor changes. We then compared the performance of the original and modified Guthrie and Agüero assays, focusing on sensitivity and specificity.

## Materials and methods

### In vitro evaluation of Guthrie and Agüero assays

#### Samples

We selected 150 samples identified as positive during routine AHSV testing using the Guthrie assay from archived samples stored at the Equine Research Centre, Faculty of Veterinary Science, University of Pretoria (ERC; Onderstepoort, South Africa). These samples were also confirmed positive on an RT-qPCR assay targeting the AHSV NS2 gene.^23^

#### Total nucleic acid extraction

We performed total nucleic acid extractions (MagMAXCORE nucleic acid purification kit, Applied Biosystems; Kingfisher 96 magnetic particle processor, ThermoFisher) according to the manufacturer’s recommendations, with slight modifications. Briefly, we added 500 µL of wash 1 and wash 2 solutions to each well of a deep-well plate. Next, we added 10 µL of proteinase K and 20 µL of magnetic beads to each well. We then added lysis buffer and binding solution at 350 µL per well. Finally, we added 100 µL of blood to each well. Negative and positive controls were added to each plate. The plate was transferred to the magnetic particle processor and processed using a modified 4462359 DW HV script (Applied Biosystems). The final 3-min nucleic acid elution step involved incubation at 95°C in 90 µL of elution buffer to denature dsRNA. The plate was immediately sealed using a foil seal and placed in a freezer at −20°C for 5 min.

#### RT-qPCR assays

The forward and reverse primer concentrations were 200 nM, and the probe concentration was 120 nM (ThermoFisher; **Table 1**). We performed RT-qPCR by adding 6.25 µL of buffer, 0.5 µL of enzyme (AgPath-ID one-step RT-PCR mastermix; Applied Biosystems), and 3.25 µL of primer/probe mix to each well of a 96-well PCR plate. We transferred 2.5 µL of the denatured eluate from the elution plate to the designated well on the PCR plate. Negative template and low- and high-positive AHSV template controls were added to each plate. We sealed the plate with a transparent plate sealer and performed RT-qPCR (QuantStudio 5 real-time PCR system; Applied Biosystems). We used the following thermoprofile: initial step 48°C for 10 min, 95°C for 10 min, 40 cycles at 95°C for 15 s, 60°C for 45 s. This annealing temperature was higher than the 55°C originally described for the Agüero assay. We classified samples as positive if the normalized fluorescence (∆Rn) for the AHSV assay exceeded 0.1 before cycle 40. If the ∆Rn for the AHSV assay did not exceed 0.1 before cycle 40, we classified the samples as negative.

**Table 1.** Sequence of the forward primer, probe, and reverse primer for the Agüero, Guthrie, Quan, modified Guthrie, and modified Agüero assays.

Assay	Target gene		Sequence
Agüero	VP7	FW (5′–3′)	CCAGTAGGCCAGATCAACAG
		Probe (5′–3′)	GCTAGCAGCCTACCACTA
		RV (5′–3′)	CTAATGAAAGCGGTGACCGT
Guthrie	VP7	FW (5′–3′)	AGAGCTCTTGTGCTAGCAGCCT
		Probe (5′–3′)	TGCACGGTCACCGCT
		RV (5′–3′)	GAACCGACGCGACACTAATGA
Quan	NS2	FW (5′–3′)	GGGAAGTGCTACRCATTACCA
		Probe (5′–3′)	TGCTGTGCTAATGAC
		RV (5′–3′)	TGCTGGGAGAATCATGTAACTC
Modified Guthrie	VP7	FW (5′–3′)	AGAGCTCTTGTGCTAGC**R**GC
		Probe (5′–3′)	TGCACGGTC**R**CCGCT
		RV (5′–3′)	GAAC**Y**GACGCGAC**R**CTA**R**TGA
Modified Agüero	VP7	FW (5′–3′)	CCAGTAGGCCAGATCAACAG
		Probe (5′–3′)	GCTAGC**R**GCYTACCACTA
		RV (5′–3′)	CTAATGAAAGCGG**Y**GACC GT

#### Sequencing

Using published terminal primers,^29^ we sequenced the VP7 gene of the 13 samples negative for AHSV detection by the Agüero assay, as well as 3 samples in which AHSV was detected by all assays. Samples were analyzed (BigDye Terminator v.3.1 cycle sequencing kit, 3130xl genetic analyzer; Applied Biosystems) according to the manufacturer’s protocol. Sequences were aligned in Geneious^18^ using MAFFT.^16,17^

### In silico analysis of the Guthrie and Agüero assays

We imported 263 AHSV VP7 gene sequences from GenBank into Geneious,^18^ and trimmed and concatenated the sequences to contain only the nucleotides complementary to the primers and probes. We aligned the 263 AHSV VP7 gene sequences with the 16 AHSV VP7 sequences described above. Using MAFFT,^16,17^ we aligned the 279 sequences against the primers and probes of the Guthrie and Agüero assays. The results were evaluated, and modifications were made to both the Guthrie assay and the Agüero assay.

### Second in vitro evaluation

#### Samples

We re-extracted and re-tested the original 150 samples identified as positive during routine AHSV testing from archived samples stored at the ERC. We also tested 32 negative surveillance samples. The 182 samples were run in parallel using the Guthrie and Agüero assays, the modified Guthrie assay, the modified Agüero assay, and the Quan NS2 assay. Methods for nucleic acid extraction and RT-qPCR are described above.

#### Comparability studies

We tested 150 AHSV-positive samples in parallel on the same RNA extracts using the Guthrie assay, the Agüero assay, the Quan et al.^23^ NS2 assay (hereafter **Quan assay**), the modified Guthrie assay, and the modified Agüero assay. Any sample with a Ct <40 was considered positive. We also tested 32 negative samples to assess the specificity of the assays. To visually assess the correlation between the different methods, we used scatter plots of the Ct values obtained with the assays. Spearman rank correlation was used to analyze the correlation between the different methods. Bland–Altman plots were used to simultaneously display and analyze the results of the different methods by plotting the difference between 2 methods against the average of 2 methods. We calculated the diagnostic sensitivity for the 4 VP7 assays by dividing the number of true-positive samples by the sum of the number of true-positive samples and false-negative samples.

## Results

### In vitro evaluation of the Guthrie and Agüero assays

Of the 150 samples tested, all were positive for AHSV using the Guthrie and the Quan assays. AHSV was not detected within 40 cycles in 13 samples using the Agüero assay ( **Suppl. Table 1** ). The diagnostic sensitivity for the Guthrie assay was 100% (95% CI: [97.6, 100%]); that of the Agüero assay was 91.3% (95% CI: [85.6, 95.3%]).

### Sequencing

We sequenced the VP7 gene of AHSV for the 13 samples in which AHSV was not detected using the Agüero assay, as well as 3 AHSV-positive samples ( **Suppl. Table 2** , GenBank accession pending). The 3 additional samples in which AHSV was detected by all assays were included to complete 2 full columns of a 96-well plate, allowing for convenient processing.

### In silico analysis of the Guthrie and the Agüero assays

**Figure 1.**
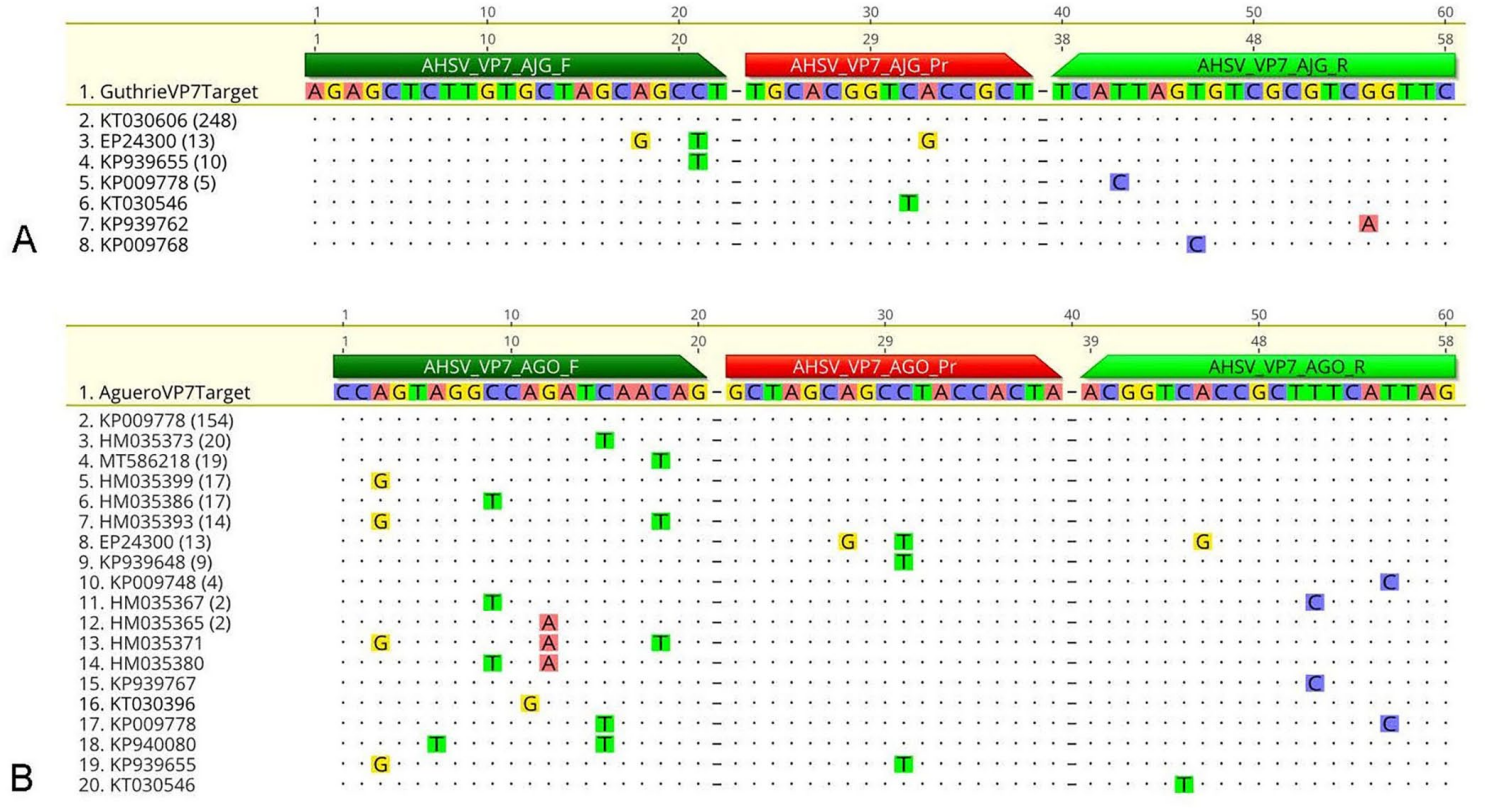
Results of in silico evaluation of the **A)** Guthrie assay, and **B)** Agüero assay alignment with 279 African horse sickness virus VP7 gene sequences, including 263 VP7 sequences downloaded from GenBank and 16 VP7 sequences from our study. Dots indicate identity with the first VP7 sequence in the group. Identical VP7 sequences are represented only once and are labeled with the accession of a randomly selected VP7 sequence in the group, followed by the number of VP7 sequences in brackets. Highlighted letters indicate a non-concordance within the VP7 sequence relative to the primer (F, R) and probe (Pr) binding site.

The Guthrie assay results (**Fig. 1A**) had 100% concordance in 248 of the 279 VP7 sequences, which included 263 sequences from GenBank, and the 16 VP7 sequences that were sequenced in our study (consensus identity 88.9%). A single nucleotide variant (**SNV**) was on the second-to-last base pair of the forward primer in 10 VP7 sequences (non-concordance 3.6%), a SNV was in the probe in 1 VP7 sequence (non-concordance 0.4%), 7 sequences with a single SNV were in 1 of 3 positions in the reverse primer (non-concordance 2.5%), and the 13 sequences that were sequenced as part of our study had 2 SNVs in the forward primer as well as 1 SNV in the probe (non-concordance 4.7%).

The Agüero assay results (**Fig. 1B**) had 100% concordance in 154 of 279 VP7 sequences (consensus identity 55.2%); 76 VP7 sequences had a single SNV in the forward primer (non-concordance 27.2%). One sequence had a single SNV in both the forward primer and a SNV in the center of the probe (non-concordance 0.4%). Nine VP7 sequences had a SNV in the center of the probe (non-concordance 3.2%). One sequence had 3 SNVs in the forward primer (non-concordance 0.4%) and 16 sequences had 2 SNVs in the forward primer (non-concordance 5.7%). Six VP7 sequences had 1 SNV in the reverse primer (non-concordance 2.1%). An additional 3 sequences had a single SNV in both the forward and reverse primers (non-concordance 1.1%). The 13 sequences that we had sequenced had 2 SNVs in the probe and 1 SNV in the reverse primer.

**Figure 2.**
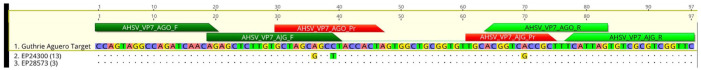
Relative African horse sickness virus (AHSV) RT-qPCR assay primer (F, R) and probe (Pr) binding sites of the Agüero and Guthrie assays, aligned with our 16 AHSV VP7 gene sequences. Two non-concordances in the Agüero probe align with 2 non-concordances at the 3′-end of the Guthrie forward primer. A single non-concordance in the Guthrie probe is in the reverse primer of the Agüero assay.

The 2 SNVs in the Agüero probe are the same 2 SNVs found at the 3′-end of the Guthrie forward primer (**Fig. 2**). A single SNV in the Guthrie assay probe is the same SNV found in the reverse primer of the Agüero assay.

### Modification of Guthrie assay

**Figure 3.**
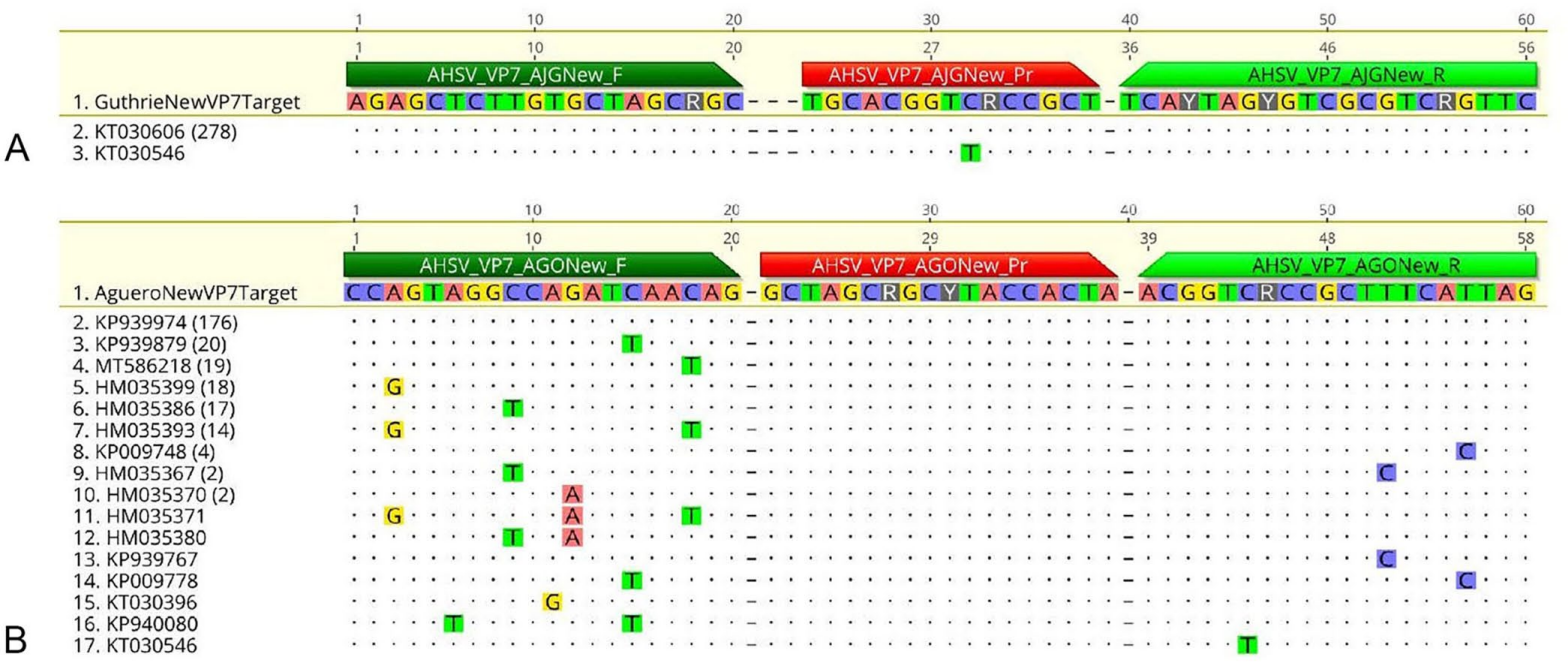
Results of in silico evaluation of the **A)** modified Guthrie African horse sickness virus (AHSV) VP7 assay, and **B)** modified Agüero VP7 assay alignment with 279 AHSV VP7 gene sequences, including 263 VP7 sequences from GenBank and our 16 VP7 sequences. Dots indicate identity with the first VP7 sequence in the group. Identical VP7 sequences are represented only once in the figure and labeled with the accession of a randomly selected VP7 sequence in the group, followed by the number of VP7 sequences in brackets. Highlighted letters indicate a non-concordance within the VP7 sequence relative to the primer (F, R) and probe (Pr) binding site.

Based on the results of the in silico evaluation of the Guthrie assay, we identified the following for modification; 1) removal of the last 2 bp at the 3′-end of the forward primer; 2) placement of a degeneracy 3 bp from the 3′-end of the forward primer; 3) placement of a degeneracy in the probe at the site where 13 sequences had a SNV; and 4) incorporation of 3 degeneracies in the reverse primer (Table 1). The amplicon length remained the same, and the melting temperature of the shortened forward primer was 60.1°C, which was lower than the 63.7°C of the original primer. We termed this the “modified Guthrie” assay. In silico analysis of the modified Guthrie assay had 99.6% concordance in the 279 VP7 sequences analyzed (**Fig. 3A**).

### Modification of the Agüero assay

Based on the results of the in silico evaluation of the Agüero assay, we addressed the issues in the 13 sequences that we had sequenced and identified the following for modification; 1) placement of 2 degeneracies in the probe, and 2) incorporation of a degeneracy in the 7th bp from the 3′-end in the reverse primer (Table 1). The amplicon length remained the same, and the melting temperature was unchanged. We termed this the “modified Agüero” assay. In silico analysis of the modified Agüero assay had 63.1% concordance in the 279 VP7 sequences analyzed (**Fig. 3B**).

Only one sequence in the silico analysis of the modified Guthrie assay (Fig. 3A) had a SNV in the probe (non-concordance 0.4%); 278 of 279 sequences had 100% concordance (99.6%). The results of the in silico analysis of the modified Agüero VP7 assay (Fig. 3B) had 100% concordance in 176 of 279 VP7 sequences (consensus identity 63.1%). No SNVs were identified in the probe.

### Second in vitro evaluation

Of the 150 samples that were re-tested, all were positive for AHSV using the Guthrie, the modified Guthrie, and the modified Agüero assays. AHSV was not detected within 40 cycles in 13 samples using the Agüero assay. In addition to the positive samples, 32 negative samples were tested using all 5 assays (Suppl. Table 1). No false positives were detected. The diagnostic sensitivity for the Guthrie, the modified Guthrie, and the modified Agüero assays were 100% (95% CI: [97.6, 100%]); sensitivity for the Agüero assay was 91.3% (95% CI: [85.6, 95.3%]). All assays had a relative specificity of 100% (95% CI: [89.1, 100%]).

### Comparability studies

**Figure 4.**
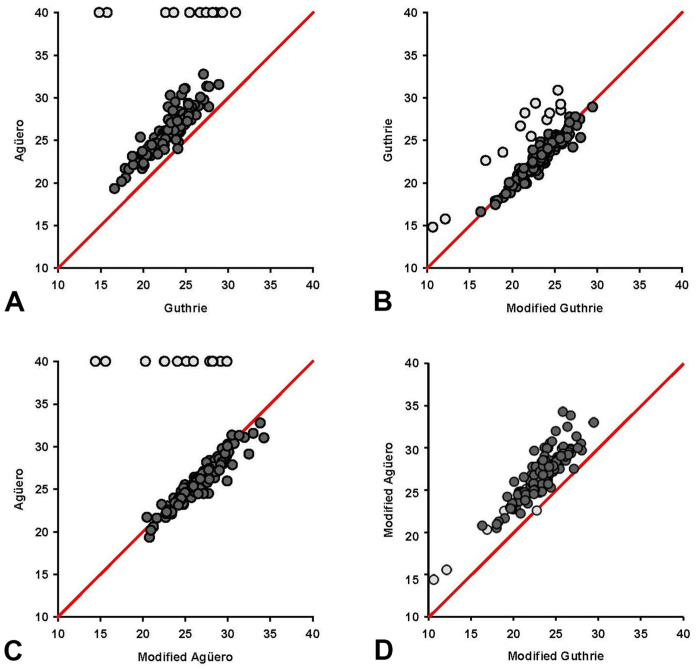
Scatter plot with equivalence line of Ct values obtained from 150 blood samples tested for African horse sickness virus (AHSV) VP7 using **A)** the Guthrie and Agüero VP7 assays, **B)** the Guthrie and modified Guthrie VP7 assays, **C)** the modified Agüero and Agüero VP7 assays, and **D)** the modified Guthrie and modified Agüero VP7 assays. Light gray dots = negative AHSV results in the Agüero VP7 assay.

We used scatter diagrams to visually compare the results obtained using the Guthrie, Agüero, modified Guthrie, and modified Agüero assays (**Fig. 4A**). The Agüero assay had consistently higher Ct values for the same sample compared with the Guthrie assay. In 13 samples, the result was positive in the Guthrie assay, but negative in the Agüero assay. A strong positive correlation was present between the Guthrie and the Agüero assays in a Spearman rank correlation analysis (*ρ* = 0.832, *p* < 0.001).

The modified Guthrie and Guthrie assay (**Fig. 4B**) results were well distributed along the diagonal line, with a few outliers with a higher Ct value for the Guthrie assay. Most of these outliers corresponded with samples that were negative in the Agüero assay. A strong positive correlation was present between the 2 assays (*ρ* = 0.863, *p* < 0.001). Results of the Agüero and the modified Agüero assays were well distributed along the diagonal lines, except for the 13 samples that were negative in the Agüero assay and positive in the modified Agüero assay (**Fig. 4C**), with a strong positive correlation between the 2 assays (ρ = 0.700, *p* < 0.001). The modified Guthrie assay had consistently lower Ct values compared with the modified Agüero assay (**Fig. 4D**), with a strong positive correlation between the assays (*ρ* = 0.881, *p* < 0.001).

**Figure 5.**
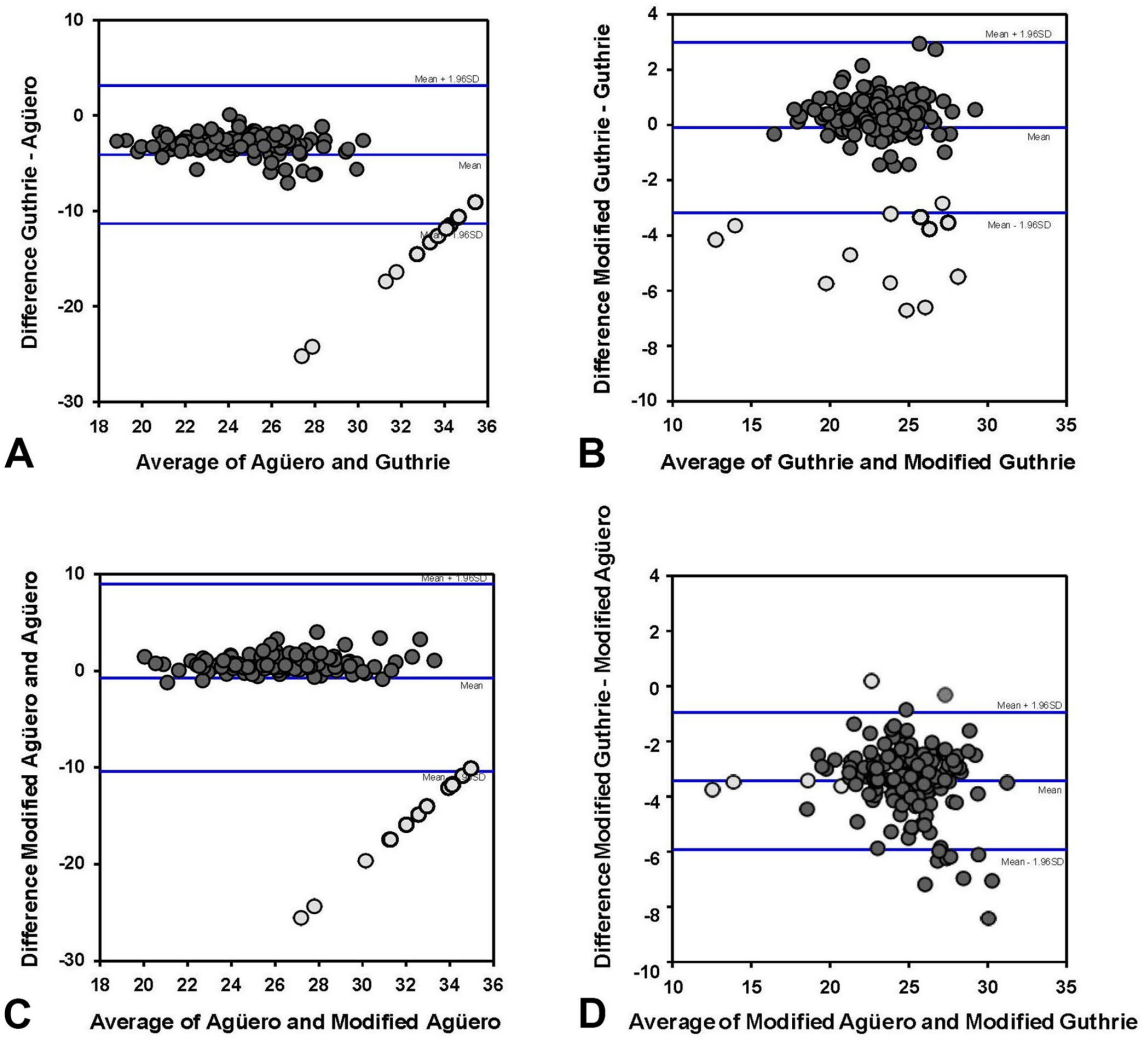
Bland–Altman plots of differences of Ct values in 4 RT-qPCR assays for African horse sickness virus: **A)** Agüero and Guthrie, **B)** Guthrie and modified Guthrie, **C)** Agüero and modified Agüero, and **D)** modified Agüero and modified Guthrie. Light gray dots = negative AHSV results in the Agüero assay.

In Bland–Altman plots ( **Suppl. Table 3** ), the mean bias ± SD was −4.07 ± 3.68 between the Agüero and Guthrie assays (**Fig. 5A**); −0.71 ± 4.95 between the Agüero and modified Agüero assays (**Fig. 5B**); 3.45 ± 1.27 between the modified Agüero and modified Guthrie assays (**Fig. 5C**); and −0.9 ± 1.57 between the Guthrie and modified Guthrie assays (**Fig. 5D**).

## Discussion

Using 150 AHSV-positive clinical samples, the Guthrie assay had a sensitivity of 100%. The Agüero assay, however, failed to detect 13 of 150 positive samples (8.7% false-negative rate). Based on these results, we sequenced the AHSV VP7 gene of the 13 sequences in which the Agüero assay failed to detect AHSV.

Based on the in silico analysis of the Guthrie assay, we identified 2 SNVs toward the 3′-end of the forward primer and an SNV in the probe with the potential to negatively influence assay sensitivity. By incorporating minor modifications in the primers and probe of this assay, we were able to achieve a consensus identity of 99.6% with the modified Guthrie assay. We did not address the SNV identified in the probe when comparing the VP7 sequence with the GenBank accession KT030546. Only one sequence had this SNV in the probe, and we did not have access to the isolate to analyze it further. Similarly, we incorporated modifications in the primer and reverse probe of the Agüero assay, increasing the consensus identity to 63%. No modifications were made to the forward primer of the Agüero assay.

The WOAH Terrestrial Manual classifies changes to a validated test method as either major or minor.^28^ A major change involves a change in the target species, a different specimen type, or change of primers and probes for different targets in the same or different genes.^28^ The modified Guthrie assay that we used can be classified as a minor change. The target region of the gene and the amplicon length remained the same. The melting temperature of the forward primer in the modified Guthrie assay was 60.1°C; that of the original Guthrie assay was 63.7°C. Although the melting temperature of the forward primer decreased, the PCR reaction is run at 60°C, which is closer to the melting temperature of the modified Guthrie assay compared with the original Guthrie assay.

Inclusion of an internal control is highly recommended in AHSV RT-qPCRs to identify samples with a false-negative result.^28^ The concentrations of the primers in the Guthrie assay are limited to support multiplexing with XenoRNA internal positive control assay.^10^ A multiplex version of the Agüero assay, with ß-actin as an internal control, is included in the WOAH manual.^28^ To allow concurrent analysis of samples on the same plate for each assay, we limited the primer concentrations for all assays, using the method detailed for the Guthrie assay. These concentrations were not individually optimized for each assay, and they may have negatively impacted the sensitivity of individual assays. The annealing temperature for the 5 assays was calculated for the primer concentration of 200 µM and was 57–62.1°C.^26^ Therefore, we used an amplification temperature of 60°C for all assays. The annealing temperature of 60°C is higher than the 55°C described for the Agüero assay, potentially reducing assay sensitivity and influencing comparative in vitro findings.

We tested 5 assays in vitro using 150 AHSV-positive and 32 AHSV-negative clinical samples. The Guthrie, modified Guthrie, modified Agüero, and Quan assays had comparable results, with sensitivities of 100%. The Agüero assay, however, failed to detect 13 of 150 positive samples (8.7% false-negative rate). False-negative results carry the risk that an animal positive for AHSV will be classified as negative and moved to a non-endemic region, which can have catastrophic consequences. The Ct values for the Guthrie and the modified Guthrie assays were very similar, with the modified Guthrie assay performing better in some of the samples. Overall, the average Ct of the modified Guthrie assay was 0.1 lower than the Guthrie assay.

The overall concordance achieved by the Agüero assay in silico was only 55.2%; in vitro analysis of the Agüero assay had an 8.7% false-negative result. Minor changes made to the probe and reverse primer only increased the consensus identity to 63% but eliminated the false-negative in vitro results. The average Ct of the modified Agüero assay was lower than that of the Agüero assay—for samples that amplified using both assays. With minor changes to the current WOAH-recommended RT-qPCR assays for the detection of AHSV, we improved assay performance; such modifications are necessary to identify novel AHSV strains.

## Supplemental Material

sj-pdf-1-vdi-10.1177_10406387261417355 – Supplemental material for Enhancing African horse sickness virus detection: comparing and adapting PCR assays Supplemental material, sj-pdf-1-vdi-10.1177_10406387261417355 for Enhancing African horse sickness virus detection: comparing and adapting PCR assays by Lisa Penzhorn, Jan E. Crafford and Alan J. Guthrie in Journal of Veterinary Diagnostic Investigation
