# Supplementary material for: Enhancing African horse sickness virus detection: comparing and adapting PCR assays
Source: J Vet Diagn Invest. 2026 Feb 7:10406387261417355. Online ahead of print. doi: 10.1177/10406387261417355 (PMC12882837; doi:10.1177/10406387261417355)
Supplement: sj-pdf-1-vdi-10.1177_10406387261417355 – Supplemental material for Enhancing African horse sickness virus detection: comparing and adapting PCR assays [file sj-pdf-1-vdi-10.1177_10406387261417355.pdf]

JVDI: Supplemental material

Penzhorn L, et al. Enhancing African horse sickness virus detection: comparing and adapting PCR assays

**Supplemental Table 1.** Ct values for 150 positive and 32 negative African horse sickness virus samples tested with the Agüero, Guthrie, modified Guthrie, modified Agüero, and Quan assays.

| | Ct values | | | | |
|---|---|---|---|---|---|
| Sample | Agüero | Guthrie | Modified Guthrie | Modified Agüero | Quan |
| EP24003 | 30.3 | 23.2 | 23.7 | 30.0 | 30.6 |
| EP24025 | 23.1 | 19.5 | 20.5 | 23.7 | 24.3 |
| EP24056 | 27.8 | 24.8 | 23.3 | 28.4 | 30.9 |
| EP24058 | 27.9 | 25.7 | 24.3 | 30.6 | 34.5 |
| EP24068 | 26.4 | 23.9 | 23.7 | 26.4 | 34.7 |
| EP24074 | 29.5 | 23.8 | 24.1 | 30.0 | 34.2 |
| EP24157 | 30.4 | 24.5 | 24.6 | 30.8 | 29.8 |
| EP24160 | 26.0 | 24.3 | 23.9 | 29.9 | 30.5 |
| EP24173 | 31.4 | 27.5 | 28.0 | 30.5 | 34.5 |
| EP24192 | 27.4 | 25.6 | 25.2 | 28.9 | 36.0 |
| EP24257 | 27.4 | 23.0 | 22.5 | 27.7 | 32.0 |
| EP24300 | ND | 26.7 | 21.0 | 24.1 | 30.8 |
| EP24364 | 25.4 | 22.5 | 23.5 | 25.7 | 32.9 |
| EP24389 | 26.0 | 24.0 | 23.7 | 27.6 | 35.3 |
| EP24404 | 23.7 | 20.3 | 20.7 | 23.9 | 32.2 |
| EP24409 | 26.4 | 23.7 | 24.0 | 26.3 | 36.3 |
| EP24414 | 23.0 | 20.0 | 21.7 | 23.4 | 26.7 |
| EP24420 | ND | 28.6 | 25.7 | 29.2 | 37.1 |
| EP24423 | ND | 30.9 | 25.4 | 28.3 | 33.1 |
| EP24442 | 21.7 | 19.9 | 20.0 | 22.7 | 27.2 |
| EP24448 | 23.2 | 20.9 | 20.6 | 24.8 | 27.7 |
| EP24470 | 40.0 | 28.2 | 21.5 | 25.1 | 29.3 |
| EP24495 | 23.8 | 20.3 | 21.0 | 24.1 | 32.8 |
| EP24507 | 26.9 | 24.2 | 24.3 | 27.7 | 29.6 |
| EP24515 | 40.0 | 25.5 | 22.2 | 26.0 | 30.1 |
| EP24576 | 27.5 | 24.6 | 24.8 | 27.9 | 35.6 |
| EP24631 | ND | 29.3 | 25.7 | 29.9 | 38.1 |
| EP24698 | ND | 27.4 | 24.1 | 27.9 | 34.8 |

| | | | | | |
|---|---|---|---|---|---|
| EP24714 | 26.4 | 23.2 | 22.9 | 26.1 | 34.6 |
| EP24750 | 25.2 | 21.6 | 22.0 | 24.9 | 29.0 |
| EP24774 | 21.7 | 17.9 | 18.0 | 20.5 | 27.7 |
| EP24778 | 27.9 | 25.5 | 25.2 | 27.8 | 35.6 |
| EP24779 | 27.8 | 24.8 | 25.1 | 28.4 | 33.8 |
| EP24781 | 28.0 | 24.6 | 25.2 | 27.6 | 35.0 |
| EP24783 | 26.2 | 23.8 | 23.3 | 26.2 | 34.6 |
| EP24789 | ND | 22.6 | 16.9 | 20.3 | 27.9 |
| EP24794 | ND | 23.6 | 18.9 | 22.5 | 29.6 |
| EP24813 | 23.7 | 21.0 | 21.2 | 24.4 | 31.5 |
| EP24815 | 28.9 | 27.7 | 26.8 | 29.9 | 34.8 |
| EP24867 | ND | 28.2 | 24.4 | 28.2 | 36.5 |
| EP24873 | 23.0 | 20.1 | 20.3 | 22.9 | 29.6 |
| EP24876 | ND | 29.4 | 22.7 | 22.6 | 28.8 |
| EP24901 | ND | 15.8 | 12.1 | 15.6 | 23.8 |
| EP24902 | ND | 14.8 | 10.7 | 14.4 | 24.2 |
| EP24943 | 31.6 | 28.9 | 29.5 | 33.0 | 34.1 |
| EP27199 | 23.2 | 20.5 | 20.8 | 22.2 | 25.9 |
| EP27224 | 25.6 | 23.5 | 23.5 | 26.0 | 31.9 |
| EP27225 | 24.5 | 21.7 | 20.9 | 24.8 | 31.9 |
| EP27243 | 28.3 | 24.4 | 24.2 | 28.1 | 34.3 |
| EP27247 | 28.1 | 24.7 | 25.2 | 28.7 | 36.0 |
| EP27284 | 22.1 | 20.1 | 20.1 | 23.4 | 29.0 |
| EP27292 | 28.9 | 23.0 | 22.4 | 29.6 | 36.3 |
| EP27302 | 25.5 | 21.8 | 23.1 | 25.0 | 30.0 |
| EP27305 | 28.3 | 25.6 | 25.6 | 28.9 | 35.7 |
| EP27326 | 21.6 | 18.3 | 19.0 | 21.6 | 27.3 |
| EP27423 | 22.6 | 19.4 | 19.6 | 22.9 | 30.3 |
| EP27432 | 24.0 | 20.9 | 20.7 | 23.7 | 29.9 |
| EP27446 | 25.2 | 22.6 | 23.3 | 26.5 | 31.5 |
| EP27459 | 29.4 | 25.3 | 25.9 | 30.1 | 35.9 |
| EP27478 | 25.8 | 23.2 | 24.1 | 26.2 | 34.0 |
| EP27483 | 28.0 | 23.9 | 25.0 | 28.5 | 34.5 |
| EP27486 | 25.1 | 22.2 | 22.9 | 27.1 | 33.7 |
| EP27501 | 31.1 | 24.9 | 25.0 | 32.0 | 35.6 |
| EP27504 | 25.4 | 23.0 | 23.3 | 26.0 | 35.5 |
| EP27517 | 28.2 | 24.6 | 25.9 | 28.9 | 34.7 |
| EP27541 | 24.4 | 21.0 | 23.1 | 24.7 | 29.7 |
| EP27558 | 25.3 | 21.6 | 22.1 | 26.8 | 32.5 |
| EP27562 | 26.1 | 24.4 | 23.3 | 27.6 | 34.8 |
| EP27575 | 27.4 | 24.6 | 24.3 | 28.0 | 36.6 |
| EP27578 | 24.8 | 21.3 | 21.1 | 24.5 | 31.5 |
| EP27581 | 25.0 | 21.8 | 22.0 | 25.9 | 30.8 |

| | | | | | |
|---|---|---|---|---|---|
| EP27599 | 23.5 | 20.7 | 21.4 | 24.0 | 31.7 |
| EP27603 | 24.5 | 21.9 | 22.0 | 25.4 | 35.6 |
| EP27605 | 25.4 | 22.7 | 23.0 | 26.2 | 34.6 |
| EP27620 | 28.4 | 24.3 | 25.1 | 27.9 | 35.6 |
| EP27633 | 24.5 | 22.1 | 22.2 | 27.7 | 34.5 |
| EP27643 | 25.2 | 21.4 | 22.8 | 25.6 | 32.9 |
| EP27649 | 28.5 | 23.5 | 23.7 | 29.0 | 35.1 |
| EP27665 | 26.1 | 23.0 | 24.2 | 26.7 | 33.6 |
| EP27688 | 24.2 | 21.5 | 21.4 | 24.7 | 33.3 |
| EP27709 | 29.1 | 26.6 | 26.6 | 29.5 | 33.4 |
| EP27713 | 24.0 | 20.5 | 21.3 | 24.2 | 27.4 |
| EP27725 | 22.3 | 19.5 | 19.8 | 23.4 | 24.9 |
| EP27738 | 25.3 | 21.3 | 22.3 | 25.7 | 29.9 |
| EP27740 | 26.1 | 22.5 | 23.0 | 26.5 | 35.8 |
| EP27754 | 26.7 | 23.6 | 24.2 | 28.5 | 30.3 |
| EP27759 | 27.0 | 24.0 | 24.2 | 27.4 | 32.2 |
| EP27816 | 27.1 | 24.6 | 24.7 | 27.2 | 35.2 |
| EP27823 | 27.8 | 24.5 | 24.7 | 28.1 | 34.9 |
| EP27839 | 29.1 | 26.0 | 26.4 | 32.5 | 36.5 |
| EP27854 | 28.8 | 25.7 | 26.3 | 28.8 | 36.0 |
| EP27957 | 28.9 | 25.7 | 26.8 | 29.7 | 34.1 |
| EP28285 | 19.3 | 16.6 | 16.3 | 20.8 | 23.6 |
| EP28286 | 24.4 | 20.8 | 21.0 | 24.8 | 27.6 |
| EP28303 | 26.2 | 24.2 | 27.2 | 27.5 | 32.2 |
| EP28307 | 29.8 | 27.2 | 26.8 | 29.4 | 34.2 |
| EP28334 | 28.3 | 25.1 | 26.2 | 28.5 | 34.4 |
| EP28573 | 25.5 | 23.6 | 23.0 | 26.8 | 33.3 |
| EP33295 | 25.3 | 22.9 | 22.7 | 26.5 | 32.7 |
| EP33513 | 26.3 | 23.8 | 23.7 | 28.5 | 34.9 |
| EP33648 | 26.1 | 21.9 | 22.6 | 27.8 | 32.7 |
| EP33861 | 24.0 | 21.2 | 21.2 | 24.7 | 29.8 |
| EP33900 | 25.8 | 22.3 | 23.9 | 26.6 | 31.7 |
| EP33957 | 22.1 | 18.8 | 19.8 | 22.7 | 27.6 |
| EP33989 | 26.7 | 23.6 | 24.4 | 27.4 | 34.9 |
| EP34004 | 25.8 | 22.6 | 23.8 | 26.1 | 31.0 |
| EP34103 | 25.5 | 22.7 | 23.4 | 25.7 | 27.3 |
| EP34200 | 23.9 | 20.4 | 20.5 | 24.4 | 32.3 |
| EP34274 | 28.2 | 24.7 | 25.5 | 27.5 | 35.4 |
| EP34275 | 24.8 | 24.2 | 24.4 | 25.3 | 28.4 |
| EP34280 | 25.4 | 22.6 | 23.7 | 26.9 | 34.4 |
| EP34292 | 28.6 | 25.5 | 26.1 | 29.0 | 35.0 |
| EP34297 | 24.0 | 24.1 | 24.1 | 25.7 | 34.0 |
| EP34337 | 26.0 | 23.2 | 23.8 | 27.4 | 29.2 |

| | | | | | |
|---|---|---|---|---|---|
| EP34342 | 25.8 | 23.2 | 23.6 | 27.6 | 30.8 |
| EP34352 | 26.8 | 24.9 | 24.8 | 27.5 | 33.6 |
| EP34576 | 26.8 | 23.8 | 24.2 | 27.0 | 34.2 |
| EP34584 | 31.0 | 24.8 | 25.8 | 34.3 | 35.9 |
| EP34610 | 28.0 | 26.2 | 26.5 | 29.5 | 36.5 |
| EP34837 | 24.7 | 21.4 | 22.0 | 26.1 | 34.5 |
| EP34857 | 25.1 | 23.9 | 22.4 | 26.8 | 31.0 |
| EP34874 | 24.5 | 22.5 | 23.1 | 27.2 | 34.1 |
| EP34882 | 23.3 | 20.4 | 21.3 | 24.7 | 28.9 |
| EP35072 | 25.2 | 22.1 | 22.4 | 25.8 | 32.0 |
| EP35194 | 27.7 | 25.0 | 25.1 | 28.4 | 35.2 |
| EP35250 | 25.2 | 22.6 | 23.4 | 24.8 | 32.6 |
| EP35483 | 23.7 | 19.9 | 21.5 | 24.5 | 29.0 |
| EP36974 | 24.6 | 21.7 | 22.5 | 25.0 | 30.3 |
| EP37051 | 26.6 | 24.4 | 24.7 | 27.8 | 31.4 |
| EP37100 | 31.3 | 27.8 | 27.4 | 31.4 | 34.0 |
| EP37140 | 24.6 | 22.3 | 22.4 | 25.0 | 29.2 |
| EP37185 | 26.2 | 23.7 | 23.5 | 27.8 | 30.6 |
| EP37186 | 24.5 | 21.0 | 21.2 | 26.5 | 30.7 |
| EP37199 | 29.2 | 25.3 | 28.1 | 29.7 | 34.4 |
| EP37244 | 30.1 | 26.8 | 27.6 | 30.0 | 34.0 |
| EP37261 | 28.2 | 25.4 | 25.8 | 29.3 | 32.5 |
| EP37275 | 32.8 | 27.1 | 26.8 | 33.8 | 34.8 |
| EP37333 | 23.9 | 22.5 | 22.4 | 24.5 | 28.5 |
| EP37372 | 23.4 | 21.7 | 21.4 | 23.8 | 28.1 |
| EP37380 | 26.2 | 22.9 | 23.5 | 26.8 | 31.2 |
| EP37382 | 27.1 | 23.3 | 23.5 | 28.5 | 32.5 |
| EP37458 | 25.4 | 19.7 | 20.1 | 26.0 | 25.8 |
| EP37477 | 20.6 | 17.9 | 18.2 | 21.3 | 26.3 |
| EP37478 | 27.4 | 24.7 | 24.7 | 27.8 | 31.9 |
| EP37489 | 23.1 | 18.7 | 19.3 | 24.2 | 29.7 |
| EP37491 | 27.9 | 25.4 | 26.5 | 29.2 | 31.0 |
| EP37495 | 20.2 | 17.5 | 18.0 | 20.9 | 28.8 |
| EP37497 | 22.3 | 20.0 | 19.7 | 22.8 | 28.5 |
| EP37498 | 27.2 | 25.2 | 25.6 | 29.0 | 34.7 |
| EP37514 | 27.3 | 24.0 | 24.2 | 27.8 | 29.3 |
| ESS240183 | ND | ND | ND | ND | ND |
| ESS240184 | ND | ND | ND | ND | ND |
| ESS240185 | ND | ND | ND | ND | ND |
| ESS240186 | ND | ND | ND | ND | ND |
| ESS240187 | ND | ND | ND | ND | ND |
| ESS240188 | ND | ND | ND | ND | ND |

| | | | | | |
|---|---|---|---|---|---|
| ESS240189 | ND | ND | ND | ND | ND |
| ESS240190 | ND | ND | ND | ND | ND |
| ESS240191 | ND | ND | ND | ND | ND |
| ESS240192 | ND | ND | ND | ND | ND |
| ESS240193 | ND | ND | ND | ND | ND |
| ESS240194 | ND | ND | ND | ND | ND |
| ESS240195 | ND | ND | ND | ND | ND |
| ESS240196 | ND | ND | ND | ND | ND |
| ESS240197 | ND | ND | ND | ND | ND |
| ESS240198 | ND | ND | ND | ND | ND |
| ESS240199 | ND | ND | ND | ND | ND |
| ESS240200 | ND | ND | ND | ND | ND |
| ESS240201 | ND | ND | ND | ND | ND |
| ESS240202 | ND | ND | ND | ND | ND |
| ESS240203 | ND | ND | ND | ND | ND |
| ESS240204 | ND | ND | ND | ND | ND |
| ESS240205 | ND | ND | ND | ND | ND |
| ESS240206 | ND | ND | ND | ND | ND |
| ESS240207 | ND | ND | ND | ND | ND |
| ESS240208 | ND | ND | ND | ND | ND |
| ESS240209 | ND | ND | ND | ND | ND |
| JSS250001 | ND | ND | ND | ND | ND |
| JSS250002 | ND | ND | ND | ND | ND |
| JSS250003 | ND | ND | ND | ND | ND |
| JSS250004 | ND | ND | ND | ND | ND |
| JSS250005 | ND | ND | ND | ND | ND |

ND = no fluorescence detected within 40 PCR cycles.

**Supplemental Table 2.** Complete coding sequences of 16 African horse sickness virus VP7 genes sequenced as part of our study.

| |
|---|
| **EP24901** |
| CGTTGTACGGGCATGCGTCACAGTGACAGATGCGAGAGTTAGTTTGGATCCAGGAGTGATGGAGACG |
| TTAGGGATTGCATTAATAGGTATAATGGTTTAACAAATCATTCGGTATCGATGAGGCCACAGACCCAAG |
| CAGAGCGAAATGAAATGTTTTTCATGTGTACTGATATGGTTTTAGCGGCATTGAACGTCCAAATTGGG |
| AATATTTCACCAGATTATGACCAGGCGTTGGCAACTGTGGGAGCCCTCGCAACAACTGAAATTCCATA |
| TAATGTTCAGGCCATGAATGACATCGTTAGAATAACGGGTCAAATGCAAACATTCGGACCAAGTAAAG |
| TGCAGACGGGACCTTATGCAGGAGCGGTTGAGGTGCAACAATCTGGCAGATATTACGTACCGCAAGG |
| TCGAACACGTGGTGGGTACATCAATTCAAATATTGCTGAAGTGTGTATGGATGCAGGTGCTGCGGGGC |
| AGGTCAATGCGCTGCTAGCCCCAAGGAGGGGGGACGCAGTCATGATCTATTTCGTTTGGAGACCGTT |
| ACGTATATTTTGTGATCCCCAAGGTGCGTCACTCGAGAGCGCTCCGGGAACTTTTGTCACCGTTGATG |
| GAGTAAATGTTGCGGCTGGAGATGTCGTCGCATGGAATACCATTGCACCAGTGAATGTCGGAAATCCT |
| GGGGCACGCAGGTCAATTTTACAATTTGAAGTGTTATGGTATACGTCCTTAGATAGATCGCTAGATACG |
| GTCCCGGAATTGGCTCCAACGCTCACAAGATGTTATGCGTATGTGTCTCCCACTTGGCACGCATTACG |
| CGCTGTCATCTTTCAGCAGATGAATATGCAGCCTATCAATCCGCCGATTTTTCCACCAACTGAAAGGA |
| ATGAAATTGTTGCATATCTTTTAGTGGCTTCCTTAGCTGATGTGTATGCGGCTTTGAGACCAGATTTCA |
| GAATGAATGGTGTGTTGCGCCAGTAGGCCAGATCAACAGAGCTCTTGTGCTAGCGGCTTACCACTAG |
| TGGCTGCGGTGTTGCACGGTCGCCGCTTTCATTAGTGTCGCGTCGGTCTTATGC |
| **EP24876** |
| CGTTGTACGGGCATGCGTCACAGTGACAGATGCGAGAGTTAGTTTGGATCCAGGAGTGATGGAGACG |
| TTAGGGATTGCAATTAATAGGTATAATGGTTTAACAAATATTCGGTATCGATGAGGCCACAGACCCAAG |
| CAGAGCGAAATGAAATGTTTTTCATGTGTACTGATATGGTTTTAGCGGCATTGAACGTCCAAATTGGG |
| AATATTTCACCAGATTATGACCAGGCGTTGGCAACTGTGGGAGCCCTCGCAACAACTGAAATTCCATA |
| TAATGTTCAGGCCATGAATGACATCGTTAGAATAACGGGTCAAATGCAAACATTCGGACCAAGTAAAG |
| TGCAGACGGGACCTTATGCAGGAGCGGTTGAGGTGCAACAATCTGGCAGATATTACGTACCGCAAGG |
| TCGAACACGTGGTGGGTACATCAATTCAAATATTGCTGAAGTGTGTATGGATGCAGGTGCTGCGGGGC |
| AGGTCAATGCGCTGCTAGCCCCAAGGAGGGGGGACGCAGTCATGATCTATTTCGTTTGGAGACCGTT |
| ACGTATATTTTGTGATCCCCAAGGTGCGTCACTCGAGAGCGCTCCGGGAACTTTTGTCACCGTTGATG |
| GAGTAAATGTTGCGGCTGGAGATGTCGTCGCATGGAATACCATTGCACCAGTGAATGTCGGAAATCCT |
| GGGGCACGCAGGTCAATTTTACAATTTGAAGTGTTATGGTATACGTCCTTAGATAGATCGCTAGATACG |
| GTCCCGGAATTGGCTCCAACGCTCACAAGATGTTATGCGTATGTGTCTCCCACTTGGCACGCATTACG |
| CGCTGTCATCTTTCAGCAGATGAATATGCAGCCTATCAATCCGCCGATTTTTCCACCAACTGAAAGGA |
| ATGAAATTGTTGCATATCTTTTAGTGGCTTCCTTAGCTGATGTGTATGCGGCTTTGAGACCAGATTTCA |
| GAATGAATGGTGTTGTTGCGCCAGTAGGCCAGATCAACAGAGCTCTTGTGCTAGCGGCTTACCACTA |
| GTGGCTGCGGTGTTGCACGGTCGCCGCTTTCATTAGTGTCGCGTCGGTCCTA |
| **EP24902** |
| GTTGTACGGGCATGCGTCACAGTGACAGATGCGAGAGTTAGTTTGGATCCAGGAGTGATGGAGACGT |
| TAGGGATTGCAATTAATAGGTATAATGGTTTAACAAATCATTCGGTATCGATGAGGCCACAGACCCAAG |
| CAGAGCGAAATGAAATGTTTTTCATGTGTACTGATATGGTTTTAGCGGCATTGAACGTCCAAATTGGG |
| AATATTTCACCAGATTATGACCAGGCGTTGGCAACTGTGGGAGCCCTCGCAACAACTGAAATTCCATA |
| TAATGTTCAGGCCATGAATGACATCGTTAGAATAACGGGTCAAATGCAAACATTCGGACCAAGTAAAG |
| TGCAGACGGGACCTTATGCAGGAGCGGTTGAGGTGCAACAATCTGGCAGATATTACGTACCGCAAGG |
| TCGAACACGTGGTGGGTACATCAATTCAAATATTGCTGAAGTGTGTATGGATGCAGGTGCTGCGGGGC |
| AGGTCAATGCGCTGCTAGCCCCAAGGAGGGGGGACGCAGTCATGATCTATTTCGTTTGGAGACCGTT |
| ACGTATATTTTGTGATCCCCAAGGTGCGTCACTCGAGAGCGCTCCGGGAACTTTTGTCACCGTTGATG |
| GAGTAAATGTTGCGGCTGGAGATGTCGTCGCATGGAATACCATTGCACCAGTGAATGTCGGAAATCCT |
| GGGGCACGCAGGTCAATTTTACAATTTGAAGTGTTATGGTATACGTCCTTAGATAGATCGCTAGATACG |
| GTCCCGGAATTGGCTCCAACGCTCACAAGATGTTATGCGTATGTGTCTCCCACTTGGCACGCATTACG |
| CGCTGTCATCTTTCAGCAGATGAATATGCAGCCTATCAATCCGCCGATTTTTCCACCAACTGAAAGGA |
| ATGAAATTGTTGCATATCTTTTAGTGGCTTCCTTAGCTGATGTGTATGCGGCTTTGAGACCAGATTTCA |
| GAATGAATGGTGTTGTTGCGCCAGTAGGCCAGATCAACAGAGCTCTTGTGCTAGCGGCTTACCACTA |
| GTGGCTGCGGTGTTGCACGGTCGCCGCTTTCATTAGTGTCGCGTCGGTCTTATGC |

**EP24470**

GTTGTACGGGCATGCGTCACAGTGACAGATGCGAGAGTTAGTTTGGATCCAGGAGTGATGGAGACGT
TAGGGATTGCAATTAATAGGTATAATGGTTTAACAAATCATTCGGTATCGATGAGGCCACAGACCCAAG
CAGAGCGAAATGAAATGTTTTTCATGTGTACTGATATGGTTTTAGCGGCATTGAACGTCCAAATTGGG
AATATTTCACCAGATTATGACCAGGCGTTGGCAACTGTGGGAGCCCTCGCAACAACTGAAATTCCATA
TAATGTTCAGGCCATGAATGACATCGTTAGAATAACGGGTCAAATGCAAACATTCGGACCAAGTAAAG
TGCAGACGGGACCTTATGCAGGAGCGGTTGAGGTGCAACAATCTGGCAGATATTACGTACCGCAAGG
TCGAACACGTGGTGGGTACATCAATTCAAATATTGCTGAAGTGTGTATGGATGCAGGTGCTGCGGGGC
AGGTCAATGCGCTGCTAGCCCCAAGGAGGGGGGACGCAGTCATGATCTATTTCGTTTGGAGACCGTT
ACGTATATTTTGTGATCCCCAAGGTGCGTCACTCGAGAGCGCTCCGGGAACTTTTGTCACCGTTGATG
GAGTAAATGTTGCGGCTGGAGATGTCGTCGCATGGAATACCATTGCACCAGTGAATGTCGGAAATCCT
GGGGCACGCAGGTCAATTTTACAATTTGAAGTGTTATGGTATACGTCCTTAGATAGATCGCTAGATACG
GTCCCGGAATTGGCTCCAACGCTCACAAGATGTTATGCGTATGTGTCTCCCACTTGGCACGCATTACG
CGCTGTCATCTTTCAGCAGATGAATATGCAGCCTATCAATCCGCCGATTTTTCCACCAACTGAAAGGA
ATGAAATTGTTGCATATCTTTTAGTGGCTTCCTTAGCTGATGTGTATGCGGCTTTGAGACCAGATTTCA
GAATGAATGGTGTTGTTGCGCCAGTAGGCCAGATCAACAGAGCTCTTGTGCTAGCGGCTTACCAMKA
GTGGCTGCGGTGTTGCACGGTCGCCGCTTTCATTAGTGTCGCGTCGGTCTTATGC

**EP24698**

GTTGTACGGGCATGCGTCACAGTGACAGATGCGAGAGTTAGTTTGGATCCAGGAGTGATGGAGACGT
TAGGGATTGCAATTAATAGGTATAATGGTTTAACAAATCATTCAGTATCGATGAGGCCACAGACCCAAG
CAGAGCGAAATGAAATGTTTTTCATGTGTACTGATATGGTTTTAGCGGCATTGAACGTCCAAATTGGG
AATATTTCACAGATTATGACCAGGCGTTGGCAACTGTGGGAGCCCTCGCAACAACTGAAATTCCATAT
AATGTTCAGGCCATGAATGACATCGTTAGAATAACGGGTCAAATGCAAACATTCGGACCAAGTAAAG
TGCAGACGGGACCTTATGCAGGAGCGGTTGAGGTGCAACAATCTGGCAGATATTACGTACCGCAAGG
TCGAACACGTGGTGGGTACATCAATTCAAATATTGCTGAAGTGTGTATGGATGCAGGTGCTGCGGGGC
AGGTCAATGCGCTGCTAGCCCCAAGGAGGGGGGACGCAGTCATGATCTATTTCGTTTGGAGACCGTT
ACGTATATTTTGTGATCCCCAAGGTGCGTCACTCGAGAGCGCTCCGGGAACTTTTGTCACCGTTGATG
GAGTAAATGTTGCGGCTGGAGATGTCGTCGCATGGAATACCATTGCACCAGTGAATGTCGGAAATCCT
GGGGCACGCAGGTCAATTTTACAATTTGAAGTGTTATGGTATACGTCCTTAGATAGATCGCTAGATACG
GTCCCGGAATTGGCTCCAACGCTCACAAGATGTTATGCGTATGTGTCTCCCACTTGGCACGCATTACG
CGCTGTCATCTTTCAGCAGATGAATATGCAGCCTATCAATCCGCCGATTTTTCCACCAACTGAAAGGA
ATGAAATTGTTGCATATCTTTTAGTGGCTTCCTTAGCTGATGTGTATGCGGCTTTGAGACCAGATTTCA
GAATGAATGGTGTTGTTGCGCCRGTAGGCCAGATCAACAGAGCTCTTGTGCTAGCGGCTTACCACTRG
TGGCTGCGGTGTTGCACGGTCGCCGCTTTCATTAGTGTCGCGTCGGTCTTATGC

**EP24631**

GTTGTACGGGCATGCGTCACAGTGACAGATGCGAGAGTTAGTTTGGATCCAGGAGTGATGGAGACGT
TAGGGATTGCAATTAATAGGTATAATGGTTTAACAAATCATTCGGTATCGATGAGGCCACAGACCCAAG
CAGAGCGAAATGAAATGTTTTTCATGTGTACTGATATGGTTTTAGCGGCATTGAACGTCCAAATTGGG
AATATTTCACCAGATTATGACCAGGCGTTGGCAACTGTGGGAGCCCTCGCAACAACTGAAATTCCATA
TAATGTTCAGGCCATGAATGACATCGTTAGAATAACGGGTCAAATGCAAACATTCGGACCAAGTAAAG
TGCAGACGGGACCTTATGCAGGAGCGGTTGAGGTGCAACAATCTGGCAGATATTACGTACCGCAAGG
TCGAACACGTGGTGGGTACATCAATTCAAATATTGCTGAAGTGTGTATGGATGCAGGTGCTGCGGGGC
AGGTCAATGCGCTGCTAGCCCCAAGGAGGGGGGACGCAGTCATGATCTATTTCGTTTGGAGACCGTT
ACGTATATTTTGTGATCCCCAAGGTGCGTCACTCGAGAGCGCTCCGGGAACTTTTGTCACCGTTGATG
GAGTAAATGTTGCGGCTGGAGATGTCGTCGCATGGAATACCATTGCACCAGTGAATGTCGGAAATCCT
GGGGCACGCAGGTCAATTTTACAATTTGAAGTGTTATGGTATACGTCCTTAGATAGATCGCTAGATACG
GTCCCGGAATTGGCTCCAACGCTCACAAGATGTTATGCGTATGTGTCTCCCACTTGGCACGCATTACG
CACTGTCATCTTTCAGCAGATGAATATGCAGCCTATCAATCCGCCGATTTTTCCACCAACTGAAAGGA
ATGAAATTGTTGCATATCTTTTAGTGGCTTCCTTAGCTGATGTGTATGCGGCTTTGAGACCAGATTTCA
GAATGAATGGTGTTGTTGCGCCAGTAGGCCAGATCAACAGAGCTCTTGTGCTAGCGGCTTACCACTA
GTGGCTGCGGTGTTGCACGGTCGCCGCTTTCATTAGTGTCGCGTCG

**EP24515**

GTTGTACGGGCATGCGTCACAGTGACAGATGCGAGAGTTAGTTTGGATCCAGGAGTGATGGAGACGT
TAGGGATTGCAATTAATAGGTATAATGGTTTAACAAATCATTCGGTATCGATGAGGCCACAGACCCAAG
CAGAGCGAAATGAAATGTTTTTCATGTGTACTGATATGGTTTTAGCGGCATTGAACGTCCAAATTGGG
AATATTTCACCAGATTATGACCAGGCGTTGGCAACTGTGGGAGCCCTCGCAACAACTGAAATTCCATA
TAATGTTCAGGCCATGAATGACATCGTTAGAATAACGGGTCAAATGCAAACATTCGGACCAAGTAAAG
TGCAGACGGGACCTTATGCAGGAGCGGTTGAGGTGCAACAATCTGGCAGATATTACGTACCGCAAGG
TCGAACACGTGGTGGGTACATCAATTCAAATATTGCTGAAGTGTGTATGGATGCAGGTGCTGCGGGGC
AGGTCAATGCGCTGCTAGCCCCAAGGAGGGGGGACGCAGTCATGATCTATTTCGTTTGGAGACCGTT
ACGTATATTTTGTGATCCCCAAGGTGCGTCACTCGAGAGCGCTCCGGGAACTTTTGTCACCGTTGATG
GAGTAAATGTTGCGGCTGGAGATGTCGTCGCATGGAATACCATTGCACCAGTGAATGTCGGAAATCCT
GGGGCACGCAGGTCAATTTTACAATTTGAAGTGTTATGGTATACGTCCTTAGATAGATCGCTAGATACG
GTCCCGGAATTGGCTCCAACGCTCACAAGATGTTATGCGTATGTGTCTCCCACTTGGCACGCATTACG
CGCTGTCATCTTTCAGCAGATGAATATGCAGCCTATCAATCCGCCGATTTTTCCACCAACTGAAAGGA
ATGAAATTGTTGCATATCTTTTAGTGGCTTCCTTAGCTGATGTGTATGCGGCTTTGAGACCAGATTTCA
GAATGAATGGTGTTGTTGCGCCAGTAGGCCAGATCAACAGAGCTCTTGTGCTAGCGGCTTACCA

**EP24423**

GTTGTACGGGCATGCGTCACAGTGACAGATGCGAGAGTTAGTTTGGATCCAGGAGTGATGGAGACGT
TAGGGATTGCAATTAATAGGTATAATGGTTTAACAAATCATTCGGTATCGATGAGGCCACAGACCCAAG
CAGAGCGAAATGAAATGTTTTTCATGTGTACTGATATGGTTTTAGCGGCATTGAACGTCCAAATTGGG
AATATTTCACCAGATTATGACCAGGCGTTGGCAACTGTGGGAGCCCTCGCAACAACTGAAATTCCATA
TAATGTTCAGGCCATGAATGACATCGTTAGAATAACGGGTCAAATGCAAACATTCGGACCAAGTAAAG
TGCAGACGGGACCTTATGCAGGAGCGGTTGAGGTGCAACAATCTGGCAGATATTACGTACCGCAAGG
TCGAACACGTGGTGGGTACATCAATTCAAATATTGCTGAAGTGTGTATGGATGCAGGTGCTGCGGGGC
AGGTCAATGCGCTGCTAGCCCCAAGGAGGGGGGACGCAGTCATGATCTATTTCGTTTGGAGACCGTT
ACGTATATTTTGTGATCCCCAAGGTGCGTCACTCGAGAGCGCTCCGGGAACTTTTGTCACCGTTGATG
GAGTAAATGTTGCGGCTGGAGATGTCGTCGCATGGAATACCATTGCACCAGTGAATGTCGGAAATCCT
GGGGCACGCAGGTCAATTTTACAATTTGAAGTGTTATGGTATACGTCCTTAGATAGATCGCTAGATACG
GTCCCGGAATTGGCTCCAACGCTCACAAGATGTTATGCGTATGTGTCTCCCACTTGGCACGCATTACG
CGCTGTCATCTTTCAGCAGATGAATATGCAGCCTATCAATCCGCCGATTTTTCCACCAACTGAAAGGA
ATGAAATTGTTGCATATCTTTTAGTGGCTTCCTTAGCTGATGTGTATGCGGCTTTGAGACCAGATTTCA
GAATGAATGGTGTTGTTGCGCCAGTAGGCCAGATCAACAGAGCTCTTGTGCTAGCGGCTTACCACTA
GTGGCTGCGGTGTTGCACGGTCGCCGCTTTCATTAGTGTCGCGTCGGTTCTTAT

**EP24420**

GTTGTACGGGCATGCGTCACAGTGACAGATGCGAGAGTTAGTTTGGATCCAGGAGTGATGGAGACST
TAGGGATTGCAATTAATAGGTATAATGGTTTAACAAATCATTCGGTATCGATGAGGCCACAGACCCAAG
CAGAGCGAAATGAAATGTTTTTCATGTGTACTGATATGGTTTTAGCGGCATTGAACGTCCAAATTGGG
AATATTTCACCAGATTATGACCAGGCGTTGGCAACTGTGGGAGCCCTCGCAACAACTGAAATTCCATA
TAATGTTCAGGCCATGAATGACATCGTTAGAATAACGGGTCAAATGCAAACATTCGGACCAAGTAAAG
TGCAGACGGGACCTTATGCAGGAGCGGTTGAGGTGCAACAATCTGGCAGATATTACGTACCGCAAGG
TCGAACACGTGGTGGGTACATCAATTCAAATATTGCTGAAGTGTGTATGGATGCAGGTGCTGCGGGGC
AGGTCAATGCGCTGCTAGCCCCAAGGAGGGGGGACGCAGTCATGATCTATTTCGTTTGGAGACCGTT
ACGTATATTTTGTGATCCCCAAGGTGCGTCACTCGAGAGCGCTCCGGGAACTTTTGTCACCGTTGATG
GAGTAAATGTTGCGGCTGGAGATGTCGTCGCATGGAATACCATTGCACCAGTGAATGTCGGAAATCCT
GGGGCACGCAGGTCAATTTTACAATTTGAAGTGTTATGGTATACGTCCTTAGATAGATCGCTAGATACG
GTCCCGGAATTGGCTCCAACGCTCACAAGATGTTATGCGTATGTGTCTCCCACTTGGCACGCATTACG
CGCTGTCATCTTTCAGCAGATGAATATGCAGCCTATCAATCCGCCGATTTTTCCACCAACTGAAAGGA
ATGAAATTGTTGCATATCTTTTAGTGGCTTCCTTAGCTGATGTGTATGCGGCTTTGAGACCAGATTTCA
GAATGAATGGTGTTGTTGCGCCAGTAGGCCAGATCAACAGAGCTCTTGTGCTAGCGGCTTACCA

| |
|---|
| **EP24409** |
| CGTTGTACGGGCATGCGTCACAGTGACAGATGCGAGAGTTAGTTTGGATCCGGGAGTAATGGAAACG<br>TTAGGGATCGCAATTAATAGGTATAATGGGTTAACAAATCATTCGGTATCGATGAGGCCGCAAACCCAA<br>GCAGAGCGAAATGAGATGTTTTTATGTGTACTGATATGGTTTTAGCGGCATTGAACGTCCAAATTGG<br>GAATATTTCACCAGATTATGACCAGGCGTTGGCAACTGTGGGAGCTCTCGCAACGACTGAAATTCCAT<br>ATAGTGTTCAGGCCATGAATGACATCGTTAGAATAACGGGCCAAATGCAAACATTCGGACCAAGTAA<br>AGTGCAGACGGGACCTTATGCAGGAGCGGCTGAGGTGCAACAATCTGGCAGATATTATGTACCGCAA<br>GGTCGAACACGTGGTGGGTACATCAATTCAAATATTGCTGAAGTGTGTATGGATGCAGGTGCTGCGGG<br>ACAGGTCAATGCGCTGCTAGCCCCAAGGAGAGGGGACGCAGTCATGATCTATTTCGTTTGGAGACCG<br>CTGCGTATATTTTGTGATCCTCAAGGTGCATCACTCGAGAGCGCTCCAGGAACTTTTGTCAACGTTGA<br>TGGAGTAAATGTTGCTGCTGGAGATGTCGTCGCATGGAATACCGTTGCGCCAGTGAATGTCGGAAAC<br>CCCGGGGCACGCAGATCAATTTTACAGTTTGAAGTGTTATGGTATACGTCCTTAGATAGATCGCTAGAC<br>ACGGTTCCGGAATTGGCTCCAACGCTCACAAGATGTTATGCGTATGTATCTCCCACTTGGCATGCATTA<br>CGCGCTGTCATTTTTCAGCAGATGAATATGCAGCCTATCAATCCGCCAATTTTTCCACCAACTGAAAG<br>GAATGAAATTGTTGCATATCTTTTAGTAGCTTCTTTAGCTGATGTGTATGCGGCTTTGAGACCAGATTTC<br>AGAATGAATGGTGTTGTTGCGCCAGTAGGCCAGATCAACAGAGCTCTTGTGCTAGCAGCCTACCACT<br>AGTGGCTGCGGTGTTGCACGGTCACCGCTTTCATTAGTGTCGCGTCGGT |
| **EP24300** |
| CGTTGTACGGGCATGCGTCACAGTGACAGATGCGAGAGTTAGTTTGGATCCAGGAGTGWTGGAGAC<br>GTTAGGGATTGCAATTAATAGGTATAATGGTTTAACAAATCATTCGGTATCGATGAGGCCACAGACCCA<br>AGCAGAGCGAAATGAAATGTTTTTCATGTGTACTGATATGGTTTTAGCGGCATTGAACGTCCAAATTG<br>GGAATATTTCACCAGATTATGACCAGGCGTTGGCAACTGTGGGAGCCCTCGCAACAACTGAAATTCC<br>ATATAATGTTCAGGCCATGAATGACATCGTTAGAATAACGGGTCAAATGCAAACATTCGGACCAAGTA<br>AAGTGCAGACGGGACCTTATGCAGGAGCGGTTGAGGTGCAACAATCTGGCAGATATTACGTACCGCA<br>AGGTCGAACACGTGGTGGGTACATCAATTCAAATATTGCTGAAGTGTGTATGGATGCAGGTGCTGCGG<br>GGCAGGTCAATGCGCTGCTAGCCCCAAGGAGGGGGGACGCAGTCATGATCTATTTCGTTTGGAGACC<br>GTTACGTATATTTTGTGATCCCCAAGGTGCGTCACTCGAGAGCGCTCCGGGAACTTTTGTCACCGTTG<br>ATGGAGTAAATGTTGCGGCTGGAGATGTCGTCGCATGGAATACCATTGCACCAGTGAATGTCGGAAAT<br>CCTGGGGCACGCAGGTCAATTTTACAATTTGAAGTGTTATGGTATACGTCCTTAGATAGATCGCTAGAT<br>ACGGTCCCGGAATTGGCTCCAACGCTCACAAGATGTTATGCGTATGTGTCTCCCACTTGGCACGCATT<br>ACGCGCTGTCATCTTTCAGCAGATGAATATGCAGCCTATCAATCCGCCGATTTTTCCACCAACTGAAA<br>GGAATGAAATTGTTGCATATCTTTTAGTGGCTTCCTTAGCTGATGTGTATGCGGCTTTGAGACCAGATT<br>TCAGAATGAATGGTGTTGTTGCGCCAGTAGGCCAGATCAACAGAGCTCTTGTGCTAGCGGCTTACCA<br>CTAGTGGCTGCGGTGTTGCACGGTCGCCGCTTTCATTAGTGTCGCGTCGGTCTTATGC |
| **EP27199** |
| GTTGTACGGGCATGCGTCACAGTGACAGATGCGAGAGTTAGTTTGGATCCAGGASTAATGGAAACGT<br>TAGGGATCGCAATCAATAGGTATAATGGGTTAACAAATCATTCGGTATCGATGAGGCCGCAAACCCAA<br>GCAGAGCGAAATGAGATGTTTTTATGTGTACTGATATGGTTTTAGCGGCATTGAACGTCCAAATTGG<br>GAATATTTCACCAGATTATGATCAGGCGTTGGCAACTGTGGGAGCTCTCGCAACGACTGAAATTCCAT<br>ATAATGTTCAGGCCATGAATGACATCGTTAGAATAACGGGCCAAATGCAAACATTCGGACCAAGTAAA<br>GTGCAGACGGGACCTTATGCAGGAGCGGCTGAGGTGCAACAATCTGGCAGATATTATGTACCGCAAG<br>GTCGAACACGTGGTGGGTACATCAATTCAAATATTGCTGAAGTGTGTATGGATGCAGGTGCTGCGGGA<br>CAGGTCAATGCGCTGCTAGCCCCAAGGAGAGGGGACGCAGTCATGATCTATTTCGTTTGGAGACCGC<br>TGCGTATATTTTGTGATCCTCAAGGTGCATCACTCGAGAGCGCTCCAGGAACTTTTGTCAACGTTGAT<br>GGAGTAAATGTTGCTGCTGGAGATGTCGTCGCATGGAATACCGTCGCGCCAGTGAATGTCGGAAACC<br>CTGGGGCACGCAGATCAATTTTACAGTTTGAAGTGTTATGGTATACGTCCTTAGATAGATCGCTAGACA<br>CGGTTCCGGAATTGGCTCCAACACTCACAAGATGTTACGCGTATGTATCTCCCACTTGGCATGCATTAC<br>GCGCTGTCATTTTTCAGCAGATGATTATGCAGCCTATCAATCCGCCAATTTTTCCACCGACTGAAAGGA<br>ATGAAATTGTTGCATATCTTTTAGTAGCTTCTTTAGCTGATGTGTATGCGGCTTTGAGACCAGATTTCAG<br>AATGAATGGTGTTGTTGCGCCAGTAGGCCAGATCAACAGAGCTCTTGTGCTAGCAGCCTACCAMKRG<br>TGGCTGCGGTGTTGCACGGTCACCGCTTTCATTAGTGTCGCGTCGGTCTTATG |

**EP24794**

CGTTGTACGGGCATGCGTCACAGTGACAGATGCGAGAGTTAGTTTGGATCCAGGAGTGATGGAGACG
TTAGGGATTGCAATTAATAGGTATAATGGTTTAACAAATCATTCGGTATCGATGAGGCCACAGACCCAA
GCAGAGCGAAATGAAATGTTTTTCATGTGTACTGATATGGTTTTAGCGGCATTGAACGTCCAAATTGG
GAATATTTCACCAGATTATGACCAGGCGTTGGCAACTGTGGGAGCCCTCGCAACAACTGAAATTCCAT
ATAATGTTCAGGCCATGAATGACATCGTTAGAATAACGGGTCAAATGCAAACATTCGGACCAAGTAAA
GTGCAGACGGGACCTTATGCAGGAGCGGTTGAGGTGCAACAATCTGGCAGATATTACGTACCGCAAG
GTCGAACACGTGGTGGGTACATCAATTCAAATATTGCTGAAGTGTGTATGGATGCAGGTGCTGCGGGG
CAGGTCAATGCGCTGCTAGCCCCAAGGAGGGGGGACGCAGTCATGATCTATTTCGTTTGGAGACCGT
TACGTATATTTTGTGATCCCCAAGGTGCGTCACTCGAGAGCGCTCCGGGAACTTTTGTCACCGTTGAT
GGAGTAAATGTTGCGGCTGGAGATGTCGTCGCATGGAATACCATTGCACCAGTGAATGTCGGAAATCC
TGGGGCACGCAGGTCAATTTTACAATTTGAAGTGTTATGGTATACGTCCTTAGATAGATCGCTAGATAC
GGTCCCGGAATTGGCTCCAACGCTCACAAGATGTTATGCGTATGTGTCTCCCACTTGGCACGCATTAC
GCGCTGTCATCTTTCAGCAGATGAATATGCAGCCTATCAATCCGCCGATTTTTCCACCAACTGAAAGG
AATGAAATTGTTGCATATCTTTTAGTGGCTTCCTTAGCTGATGTGTATGCGGCTTTGAGACCAGATTTC
AGAATGAATGGTGTTGTTGCGCCAGTAGGCCAGATCAACAGAGCTCTTGTGCTAGCGGCTTACCACK
RGTGGCTGCGGTGTTGCACGGTCGCCGCTTTCATTAGTGTCGCGTC

**EP24789**

CGTTGTACGGGCATGCGTCACAGTGACAGATGCGAGAGTTAGTTTGGATCCAGGAGTGATGGAGACG
TTAGGGATTGCAATTAATAGGTATAATGGTTTAACAAATCATTCGGTATCGATGAGGCCACAGACCCAA
GCAGAGCGAAATGAAATGTTTTTCATGTGTACTGATATGGTTTTAGCGGCATTGAACGTTCAAATTGG
GAATATTTCACCAGATTATGACCAGGCGTTGGCAACTGTGGGAGCCCTCGCAACAACTGAAATTCCAT
ATAATGTTCAGGCCATGAATGACATCGTTAGAATAACGGGTCAAATGCAAACATTCGGACCAAGTAAA
GTGCAGACGGGACCTTATGCAGGAGCGGTTGAGGTGCAACAATCTGGCAGATATTACGTACCGCAAG
GTCGAACACGTGGTGGGTACATCAATTCAAATATTGCTGAAGTGTGTATGGATGCAGGTGCTGCGGGG
CAGGTCAATGCGCTGCTAGCCCCAAGGAGGGGGGACGCAGTCATGATCTATTTCGTTTGGAGACCGT
TACGTATATTTTGTGATCCCCAAGGTGCGTCACTCGAGAGCGCTCCGGGAACTTTTGTCACCGTTGAT
GGAGTAAATGTTGCGGCTGGAGATGTCGTCGCATGGAACACCATTGCACCAGTGAATGTCGGAAATC
CTGGGGCACGCAGGTCAATTTTACAATTTGAAGTGTTATGGTATACGTCCTTAGATAGATCGCTAGATA
CGGTCCCGGAATTGGCTCCAACGCTCACAAGATGTTATGCGTATGTGTCTCCCACTTGGCACGCATTA
CGCGCTGTCATCTTTCAGCAGATGAATATGCAGCCTATCAATCCGCCGATTTTTCCACCAACTGAAAG
GAATGAAATTGTTGCATATCTTTTAGTGGCTTCCTTAGCTGATGTGTATGCGGCTTTGAGACCAGATTT
CAGAATGAATGGTGTTGTTGCGCCAGTAGGCCAGATCAACAGAGCTCTTGTGCTAGCGGCTTACCAC
TAGTGGCTGCGGTGTTGCACGGTCGCCGCTTTCATTAGTGTCGCGTC

**EP28573**

GTTGTACGGGCATGCGTCACAGTGACAGATGCGAGAGTTAGTTTGGATCCGGAGTAATGGAGACGTT
AGGGATCGCGATTAATAGATATAATGGGTTAACAAATCATTCGGTATCAATGAGGCCGCAAACCCAAG
TAGAACGAAATGAAATGTTTTTTATGTGTACTGATATGGTTTTAGCGGCATTGAACGTCCAAATTGGGA
ATATTTCACCAGATTATGATCAGGCGTTGGCAACTGKGGGRRSYYYCGCAACGACTGAAATTCCATAT
AATGTTCAGGCTATGAATGACATCGTTAGAATAACGGGCCAGATGCAAACATTCGGACCAAGTAAAGT
ACAGACGGGACCTTATGCAGGAGCGGCTGAGGTACAACAATCTGGCAGATATTATGTACCGCAAGGT
CGAACACGTGGTGGGTACATCAATTCTAATATTGCTGAAGTGTGTATGGATGCAGGTGCTGCGGGACA
GGTCAATGCGCTGCTAGCCCCAAGAAGGGGGGACGCAGTCATGATCTATTTCGTTTGGAGACCGCTG
CGTATATTTTGTGATCCTCAAGGTGCATCACTCGAGAGCGCTCCAGGAACTTTTGTCAACGTTGATGG
AGTAAATGTTGCGGCTGGAGATGTCGTCGCATGGAATACCATCGCACCAGTGAATGTCGGAAATCCA
GGGGCACGCAGATCAATTTTACAGTTTGAAGTGTTATGGTATACATCCTTAGACAGATCGCTAGACAC
GGTTCCGGAATTGGCTCCAACGCTCACAAGATGTTATGCGTATGTGTCTCCCACTTGGCACGCATTAC
GCGCTGTCATTTTTCAGCAGATGAATATGCAGCCTATCAATCCGCCAATTTTTCCACCGACTGAAAGG
AATGAAATTGTTGCGTATCTTTTGGTAGCTTCTTTAGCTGATGTATATGCGGCTTTGAGACCAGATTTCA
GAATGAATGGTGTTGTTGCGCCAGTAGGCCAGATCAACAGAGCTCTTGTGCTAGCAGCC

| **EP24867** |
|---|
| GTTGTACGGGCATGCGTCACAGTGACAGATGCGAGAGTTAGTTTGGATCCAGGAGTGATGGAGACGT<br>TAGGGATTGCAATTAATAGGTATAATGGTYTAACAAATCATTCGGTATCGATGAGGCCACAGACCCAA<br>GCAGAGCGAAATGAAATGTTTTTCATGTGTACTGATATGGTTTTAGCGGCATTGAACGTCCAAATTGG<br>GAATATTTCACCAGATTATGACCAGGCGTTGGCAACTGTGGGAGCCCTCGCAACAACTGAAATTCCAT<br>ATAATGTTCAGGCCATGAATGACATCGTTAGAATAACGGGTCAAATGCAAACATTCGGACCAAGTAAA<br>GTGCAGACGGGACCTTATGCAGGAGCGGTTGAGGTGCAACAATCTGGCAGATATTACGTACCGCAAG<br>GTCGAACACGTGGTGGGTACATCAATTCAAATATTGCTGAAGTGTGTATGGATGCAGGTGCTGCGGGG<br>CAGGTCAATGCGCTGCTAGCCCCAAGGAGGGGGGACGCAGTCATGATCTATTTCGTTTGGAGACCGT<br>TACGTATATTTTGTGATCCCCAAGGTGCGTCACTCGAGAGCGCTCCGGGAACTTTTGTCACCGTTGAT<br>GGAGTAAATGTTGCGGCTGGAGATGTCGTCGCATGGAATACCATTGCACCAGTGAATGTCGGAAATCC<br>TGGGGCACGCAGGTCAATTTTACAATTTGAAGTGTTATGGTATACGTCCTTAGATAGATCGCTAGATAC<br>GGTCCCGGAATTGGCTCCAACGCTCACAAGATGTTATGCGTATGTGTCTCCCACTTGGCACGCATTAC<br>GCGCTGTCATCTTTCAGCAGATGAATATGCAGCCTATCAATCCGCCGATTTTTCCACCAACTGAAAGG<br>AATGAAATTGTTGCATATCTTTTAGTGGCTTCCTTAGCTGATGTGTATGCGGCTTTGAGACCAGATTTC<br>AGAATGAATGGTGTTGTTGCGCCAGTAGGCCAGATCAACAGAGCTCTTGTGCTAGCGGCTTACCACT<br>AGTGGCTGCGGTGTTGCACGGTCGCCGCTTTCATTAGTGTCGCGTCGGTCTTATG |

**Supplemental Table 3.** Bland–Altman statistical summary for the 4 RT-qPCR African horse sickness virus assays.

| Assays | Estimate | 95% CI |
|---|---|---|
| **A.** Agüero and Guthrie | | |
| Bias (*n* = 150) | –4.0703 | [–4.6598, –3.4809] |
| SD | 3.6835 | |
| LL of agreement | –11.2900 | [–12.311, –10.269] |
| UL of agreement | 3.1493 | [2.1283, 4.1703] |
| **B.** Agüero and modified Agüero | | |
| Bias (*n* = 150) | –0.7062 | [–1.4991, 0.0867] |
| SD | 4.9545 | |
| LL of agreement | –10.4170 | [–11.7903, –9.0437] |
| UL of agreement | 9.0046 | [7.6313, 10.3779] |
| **C.** modified Agüero and modified Guthrie | | |
| Bias (*n* = 150) | –3.4500 | [–3.6533, –3.2467] |
| SD | 1.2706 | |
| LL of agreement | –5.9404 | [–6.2926, –5.5882] |
| UL of agreement | –0.9596 | [–1.3118, –0.6074] |
| **D.** Guthrie and modified Guthrie | | |
| Bias (*n* = 150) | –0.859 | [–0.3382, 0.1665] |
| SS | 1.5768 | |
| LL of agreement | –3.1764 | [–3.6135, –2.7394] |
| UL of agreement | 3.0047 | [2.5676, 3.4418] |

LL = lower limit; UL = upper limit.
